# The landscape of miRNA-mRNA regulatory network and cellular sources in inflammatory bowel diseases: insights from text mining and single cell RNA sequencing analysis

**DOI:** 10.3389/fimmu.2024.1454532

**Published:** 2024-08-22

**Authors:** Yuan Li, Yao Wang, Simeng Chen, Lijia Liu

**Affiliations:** Department of General Surgery, The First Affiliated Hospital of Nanjing Medical University, Nanjing, China

**Keywords:** text mining, inflammatory bowel diseases, Crohn’s disease, ulcerative colitis, single cell RNA sequencing, miRNA-mRNA regulatory network

## Abstract

**Background:**

Inflammatory Bowel Diseases (IBDs), encompassing Ulcerative Colitis (UC) and Crohn’s Disease (CD), are chronic, recurrent inflammatory conditions of the gastrointestinal tract. The microRNA (miRNA) -mRNA regulatory network is pivotal in the initiation and progression of IBDs. Although individual studies provide valuable insights into miRNA mechanisms in IBDs, they often have limited scope due to constraints in population diversity, sample size, sequencing platform variability, batch effects, and potential researcher bias. Our study aimed to construct comprehensive miRNA-mRNA regulatory networks and determine the cellular sources and functions of key miRNAs in IBD pathogenesis.

**Methods:**

To minimize potential bias from individual studies, we utilized a text mining-based approach on published scientific literature from PubMed and PMC databases to identify miRNAs and mRNAs associated with IBDs and their subtypes. We constructed miRNA-mRNA regulatory networks by integrating both predicted and experimentally validated results from DIANA, Targetscan, PicTar, Miranda, miRDB, and miRTarBase (all of which are databases for miRNA target annotation). The functions of miRNAs were determined through gene enrichment analysis of their target mRNAs. Additionally, we used two large-scale single-cell RNA sequencing datasets to identify the cellular sources of miRNAs and the association of their expression levels with clinical status, molecular and functional alternation in CD and UC.

**Results:**

Our analysis systematically summarized IBD-related genes using text-mining methodologies. We constructed three comprehensive miRNA-mRNA regulatory networks specific to IBD, CD, and UC. Through cross-analysis with two large-scale scRNA-seq datasets, we determined the cellular sources of the identified miRNAs. Despite originating from different cell types, hsa-miR-142, hsa-miR-145, and hsa-miR-146a were common to both CD and UC. Notably, hsa-miR-145 was identified as myofibroblast-specific in both CD and UC. Furthermore, we found that higher tissue repair and enhanced glucose and lipid metabolism were associated with hsa-miR-145 in myofibroblasts in both CD and UC contexts.

**Conclusion:**

This comprehensive approach revealed common and distinct miRNA-mRNA regulatory networks in CD and UC, identified cell-specific miRNA expressions (notably hsa-miR-145 in myofibroblasts), and linked miRNA expression to functional alterations in IBD. These findings not only enhance our understanding of IBD pathogenesis but also offer promising diagnostic biomarkers and therapeutic targets for clinical practice in managing IBDs.

## 1 Introduction

Inflammatory Bowel Diseases (IBDs), primarily comprising Ulcerative Colitis (UC) and Crohn’s Disease (CD), are archetypal immune-mediated inflammatory disorders affecting the gastrointestinal tract. The enigmatic etiology, limited therapeutic efficacy, and heterogeneous patient presentations underscore the significant global health burden posed by IBDs (1–4). These conditions share fundamental pathophysiological features, including dysregulated immune responses, genetic predisposition, disruption of the epithelial barrier, and alterations in gut microbiota composition (5–8). Despite these commonalities, CD and UC exhibit distinct characteristics in terms of affected gastrointestinal locations, pathological manifestations, and intestinal wall layers involved (9). The complex nature of IBDs necessitates more detailed and comprehensive molecular pathological research at cellular and molecular levels. In this context, microRNAs (miRNAs)-mRNA regulatory networks have emerged as crucial players in orchestrating cellular responses to physiological perturbations and disease conditions, including IBDs (10, 11). These small, non-coding RNAs mediate post-transcriptional gene silencing and have demonstrated indispensable roles in various cellular processes (12). For instance, miR-31 mimic alleviated the colon inflammatory response of DSS-colitis mice (13). Several mRNAs have been identified as crucial players in IBD pathogenesis. For instance, increased expression of pro-inflammatory cytokine mRNAs such as TNF-α, IL-1β, and IL-6 has been observed in IBD patients (14, 15). Additionally, mRNAs encoding proteins involved in epithelial barrier function, such as tight junction proteins (e.g., claudins and occludins), have been found to be dysregulated in IBD (16, 17). Studies have also highlighted the altered expression of mRNAs related to immune cell function, including T cell differentiation factors like STAT3 and GATA3 (18, 19). Understanding these mRNA alterations is crucial for comprehending the complex molecular landscape of IBD. Recent advancements in IBD research as well as deep learning and bioinformatics, such as the generation of large-scale single-cell RNA sequencing (scRNA-seq) datasets, have provided unprecedented opportunities to elucidate the intricate molecular mechanisms underlying these diseases. These technological advances enable a more nuanced understanding of the role of miRNAs in IBD pathogenesis and potential therapeutic interventions.

Text-mining has emerged as a powerful tool in biomedical research, offering researchers the ability to extract valuable information from vast amounts of scientific literature (20–22). This computational approach allows for the systematic analysis of large-scale textual data, providing a comprehensive view of complex biological systems and relationships. The text-mining process typically involves several key steps. First, document retrieval gathers relevant texts from databases such as PubMed or Web of Science. Next, natural language processing (NLP) techniques are applied to preprocess the text, including tokenization, part-of-speech tagging, and named entity recognition. Information extraction then identifies specific entities (e.g., genes, proteins, diseases) and their relationships. Finally, data mining techniques are used to discover patterns and generate insights from the extracted information. Text-mining has been successfully applied to various biomedical and clinical research, including potential drug-target interactions, predict adverse drug reactions, predict new cancer driver genes, and constructing large-scale molecular interaction networks (20–23). In the context of miRNA-mRNA regulatory networks, this approach can help identify potential interactions, regulatory pathways, and functional implications that might otherwise be overlooked (24). The systematic nature of text-mining also reduces bias in literature analysis, as it considers a broader range of sources and perspectives.

However, due to biases in individual studies, the comprehensive miRNA-mRNA regulatory network in IBDs is not fully understood, particularly regarding the cellular sources of specific miRNAs. In this study, we systematically summarized IBD-related miRNAs and mRNAs using a text mining approach. We constructed three comprehensive miRNA-mRNA networks for IBD, CD, and UC, respectively, through additional miRNA target gene annotation. To further elucidate these networks at the cellular and molecular levels, we employed two large-scale scRNA-seq datasets from CD and UC colon tissues to clarify the specific cell types (cellular sources) that express relevant miRNAs, as well as to clarify the molecular and functional alterations associated with these miRNAs. In conclusion, this comprehensive approach provides generalizable insights into the miRNA-mRNA regulatory network and the cellular specificity of miRNAs, offering promising diagnostic biomarkers and therapeutic targets for clinical practice in managing IBDs.

## 2 Materials and methods

### 2.1 Study design

**Figure 1:**
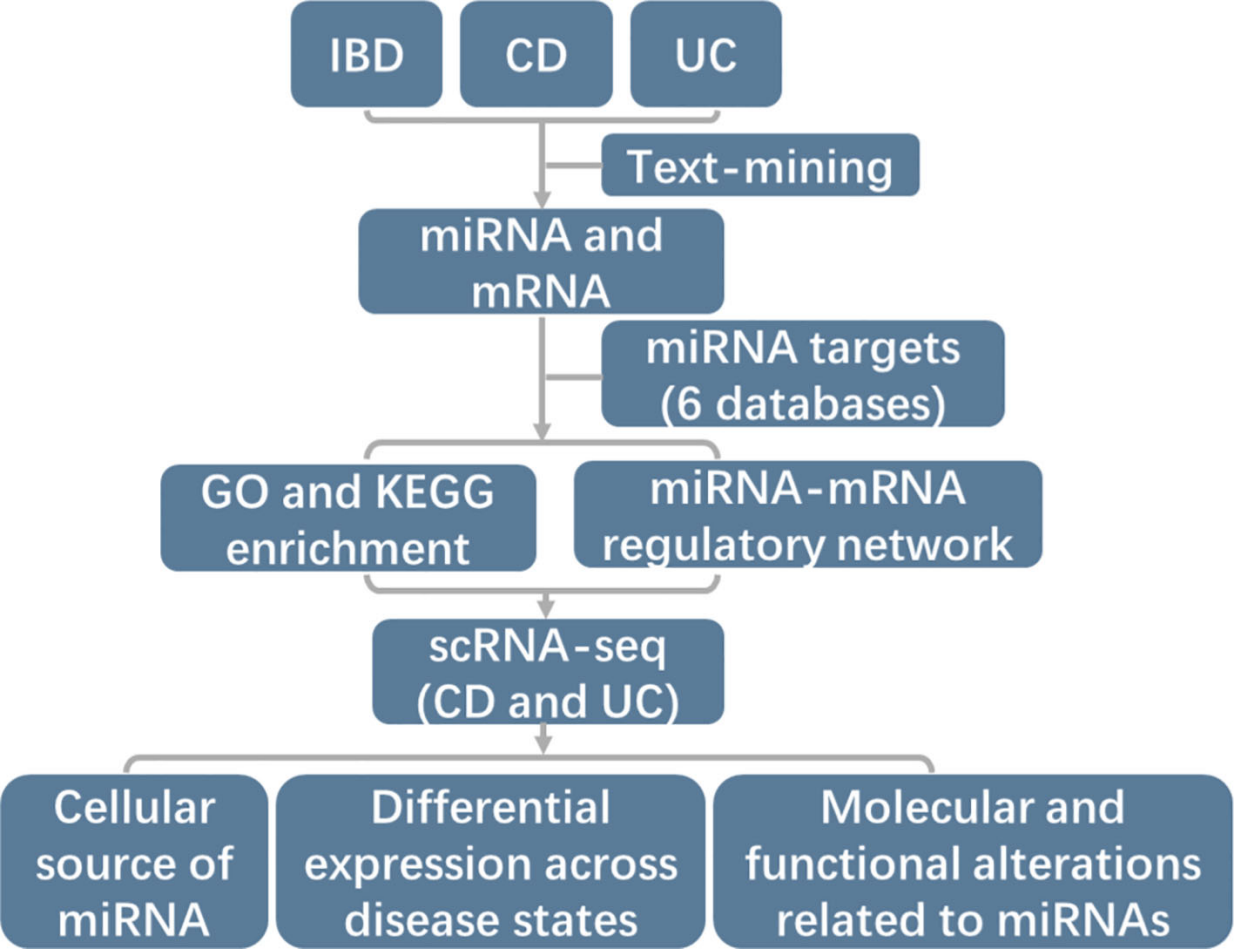
Schematic representation of the study design workflow. Firstly, IBD-associated miRNAs and mRNAs were identified by text-mining. Secondly, target mRNAs for each identified miRNA were annotated using 6 databases (DIANA, Targetscan, PicTar, Miranda, miRDB, and miRTarBase). The miRTarBase was the experimentally validated microRNA-target interactions database while the others were mostly based on prediction. Then comprehensive miRNA-mRNA regulatory networks were constructed and the function of miRNAs were determined by GO and KEGG enrichment. Lastly, two large-scale scRNA-seq datasets of CD and UC were advanced employed to examine the cell type-specific expression patterns of miRNAs, expression level across disease states and impact of miRNA regulation on cellular processes and functions in IBD.

This study aimed to construct comprehensive miRNA-mRNA regulatory networks in IBD, CD and UC through text-mining and annotated miRNA target genes (**Figure 1**). Additionally, we utilized two large-scale scRNA-seq datasets to elucidate the cellular sources of identified miRNAs, providing more specific regulatory mechanisms of miRNA-mRNA networks. We further examined the expression levels across different disease states and molecular alterations in source cells (**Figure 1**).

### 2.2 Text Mining of IBD-related miRNAs and mRNAs

IBD-related miRNAs and mRNAs were identified using PubTator 3.0 (25), which provides over one billion entity and relation annotations across approximately 36 million PubMed abstracts and 6 million full-text articles from the PMC open access subset. The search criteria employed for IBDs were “inflammatory bowel diseases,” “Crohn’s disease,” and “ulcerative colitis.” Human-origin genes were selected for further analysis and determined using the R package biomaRt (26). Genes were translated and annotated into biotypes using biomaRt.

### 2.3 Construction of miRNA-mRNA regulatory network and gene enrichment analysis

The miRNA-mRNA regulatory network was constructed in two steps. First, target mRNAs of identified miRNAs were annotated from five online databases: DIANA, TargetScan, PicTar, miRanda, and miRDB by R package miRNAtap (27–31). Only mRNAs annotated in at least two databases were included for further analysis to increase accuracy. We further cross validated the miRNA-mRNA interaction by another experimentally validated database (miRTarBase) to provide more rigorous information (32). Next, the annotated target mRNAs were further filtered by intersecting with text-mining identified mRNAs. The miRNA-mRNA regulatory network visualization was accomplished using Gephi software. Gene Ontology (GO) and Kyoto Encyclopedia of Genes and Genomes (KEGG) enrichment analyses of miRNA-targeted mRNAs were conducted using the R package clusterProfiler (33).

### 2.4 ScRNA-seq analysis

#### 2.4.1 ScRNA-seq data download, preprocessing, and cell annotation

CD and UC scRNA-seq datasets were obtained from the Single Cell Portal (https://singlecell.broadinstitute.org/single_cell) using accession numbers SCP1884 and SCP259, respectively. Comprehensive details regarding the scRNA-seq data, including clinical sample information and sequencing protocols, are available in the original source studies (33, 34). Both datasets were processed using the same criteria. Epithelial, stromal, and immune cells were downloaded and subjected to scRNA-seq analysis. Quality control removed cells with fewer than 250 expressed genes, 100 expression counts, or >25% mitochondrial reads. The scRNA-seq data were normalized, scaled, and dimensionally reduced using Principal Component Analysis (PCA) and t-distributed Stochastic Neighbor Embedding (t-SNE). We set dim=30 in the “FindNeighbors” function to construct the nearest-neighbor graph based on the standard deviations of the principal components, visualized by the “ElbowPlot” function. Cells were clustered using the “FindClusters” function with resolution=0.4. RunTSNE was applied for visualization. Cell types were primarily determined by annotated cell types from original articles. Detailed cell marker genes were obtained from previous research. The R package Seurat (Version 4.1.1) was used for this analysis (35).

#### 2.4.2 Cellular source of miRNAs

The spatial distribution of specific miRNAs was determined based on the diagnostic odds ratio (DOR) (36). The DOR for each miRNA was calculated by binarizing expression values, treating any detection of a miRNA (normalized expression value > 0) as positive and zero expression as negative. A pseudocount of 0.5 was included to avoid undefined values:


$$DOR=\frac{\left(\text{TP }+0.5\;\right)/\left(\text{FP}+0.5\right)}{\left(FN+0.5\;\right)\;/\left(TN+0.5\right)}$$

Where TP (True Positives) represents the number of cells within the group expressing the miRNA, FP (False Positives) represents cells outside the group expressing the miRNA, FN (False Negatives) represents cells within the group with no detected expression, and TN (True Negatives) represents cells outside the group with no detected miRNA expression. To reduce false positives, miRNAs expressed in fewer than 5% of cells in a subtype were considered non-significant.

#### 2.4.3 Cell subcluster analysis

Cell subclusters were determined based on the expression levels of relevant miRNAs. Differentially Expressed Genes (DEGs) between cell subclusters were identified using the “FindMarkers” function. Genes with p-value< 0.05 and log2FoldChange > 1 were considered DEGs. Subsequent GO and KEGG enrichment analyses were applied using the R package clusterProfiler. Gene set scores were calculated using the AddModuleScore function of the Seurat package (37). Hallmark gene sets were downloaded from the GSEA website (https://www.gsea-msigdb.org/gsea/index.jsp).

## 3 Results

### 3.1 miRNAs and mRNAs discovery profile in IBDs

**Figure 2:**
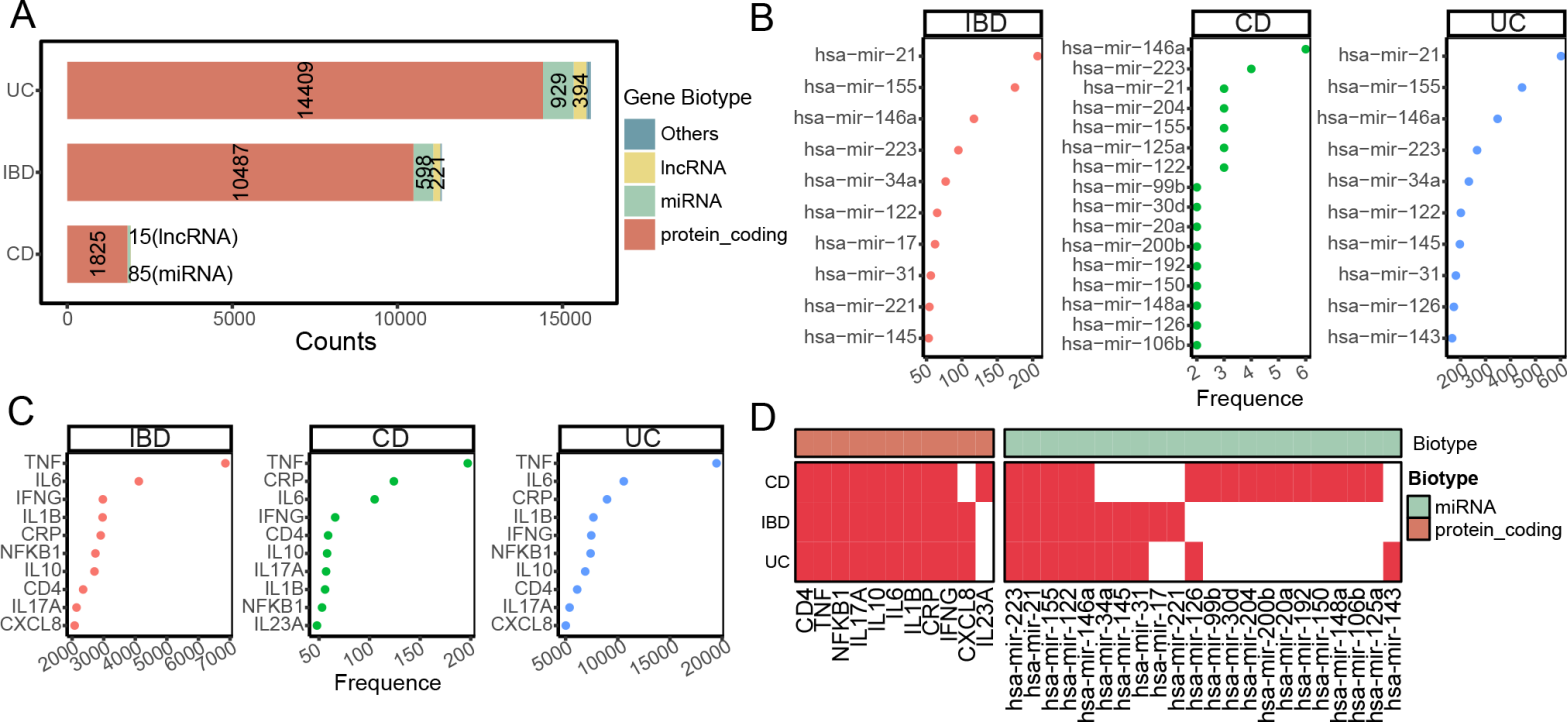
Text mining discovery profile of IBD-related miRNAs and mRNAs. **(A)** Bar plot showing the distribution of text-mining identified IBD-related genes, categorized by gene biotype. The color represents the gene biotype, majorly include protein coding, miRNA, long non-coding RNA (lncRNA). The number represents the relative biotype gene counts. **(B, C)** Dot plots illustrating the top 10 most frequently mentioned miRNAs **(B)** and mRNAs **(C)** in IBD, CD, and UC literature. The x axis represents the mentation frequency by text-mining. **(D)** Heatmap depicting the presence (red) or absence (white) of the top 10 most frequent miRNAs and mRNAs in IBD and its subtypes, as identified by text mining.

To elucidate IBD-related genes, we employed a text mining approach (**Figure 1**). We identified 11,343, 1,927, and 15,859 genes for IBD, CD, and UC, respectively (**Figure 2A**). Protein-coding genes constituted the majority (>90%) in all three IBD cohorts, with miRNAs ranking second (**Figure 2A**). The most frequently mentioned miRNAs were hsa-miR-21 for IBD and UC, and hsa-miR-146a for CD (**Figure 2B**). Consistent with previous research, the most frequently mentioned mRNAs in IBDs were inflammatory factors, including TNF, IL6, IL1B, and IFNG (**Figure 2C**). Interestingly, while the top ten mentioned mRNAs largely overlapped among IBDs, the top miRNAs showed more frequent overlap between IBD and UC than CD (**Figure 2D**). Notably, both miRNAs and mRNAs identified in CD were less frequent compared to UC (**Figures 2A–C**), indicating that CD has been less studied.

### 3.2 Constructed miRNA-mRNA regulatory network in IBDs

**Figure 3:**
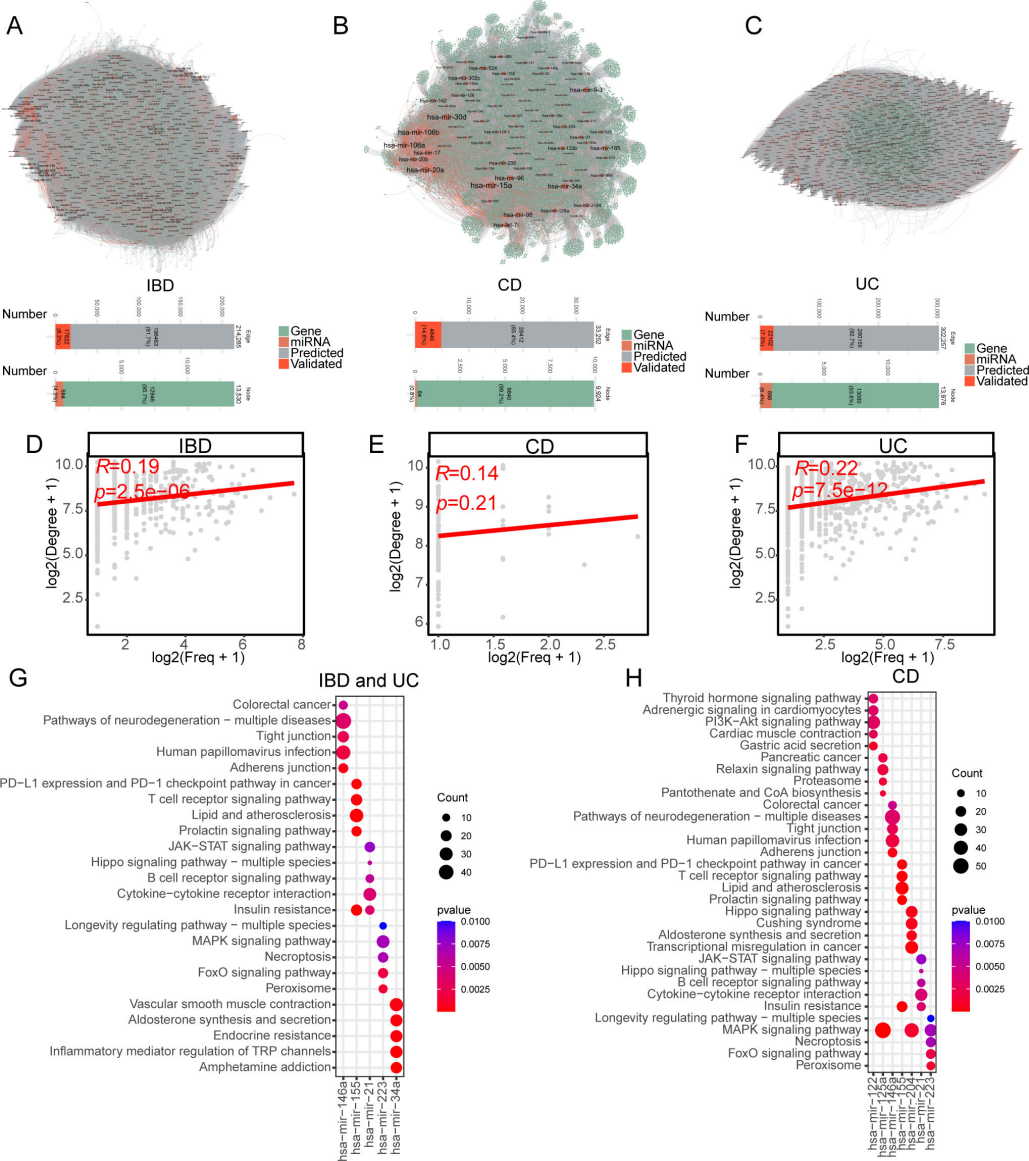
Comprehensive miRNA-mRNA regulatory networks in IBD, CD, and UC. **(A–C)** Visualization of miRNA-mRNA regulatory networks in IBD **(A)**, CD **(B)**, and UC **(C)**. The node color represents the miRNA (orange) and mRNA (green). The edge color represents the support type of miRNA-mRNA interaction. The grey edge between nodes represents the predicted miRNA-mRNA interaction while the red represents the experimentally validated miRNA-mRNA interaction. The bottom chart represents the network statistic of edge and node number. The edge number barplot was colored by predicted or experimentally validated miRNA-mRNA interaction. The node number barplot was colored by gene biotype of miRNA or mRNA. **(D–F)** Scatter plots showing the correlation between miRNA text-mining annotation frequency and degree in miRNA-mRNA networks for IBD **(D)**, CD **(E)**, and UC **(F)**. The x axis represents the log-transformed miRNA annotation frequency by text mining while the y axis represents the log-transformed miRNA degree in miRNA-mRNA regulatory network. Spearman correlation coefficients and p-values are indicated in the upper left corner of each plot. **(G, H)** Top 5 KEGG enrichment terms for the highest-ranked miRNAs in IBD **(G)**, UC **(G)**, and CD **(H)**. miRNA rankings were determined by the average of text-mining annotation frequency and degree in the miRNA-mRNA network.

To construct IBD subtype-specific miRNA-mRNA regulatory networks, we integrated text-mining identified miRNAs/mRNAs with predicted miRNA-mRNA pairs from six online databases (**Figure 1**). By integrating both predicted and experimentally validated miRNA databases, we constructed miRNA-mRNA regulatory networks of IBD, CD and UC. The resulting regulatory networks contain 584 miRNAs and 12,946 mRNAs for IBD, 84 miRNAs and 9,840 mRNAs for CD, and 896 miRNAs and 13,080 mRNAs for UC (**Figures 3A–C**). As for miRNA-mRNA interactions (edges in network), 214,285 (91.7% predicted and 8.3% validated) edges were identified in IBD while 33,252 (85.4% predicted and 14.5% validated) and 302,257(92.7% predicted and 7.3% validated) edges were identified in CD and UC, respectively (**Figures 3A–C**). The miRNAs in the regulatory network demonstrated a significant Spearman correlation between text-mining mentioned frequency and degree of regulatory network in both IBD (r=0.19, p=2.5e-6) and UC (r=0.22, p=7.5e-12), suggesting that well-studied miRNAs are likely involved in regulating a wider range of biological processes (**Figures 3D–F**). Although not statistically significant, CD also showed a positive correlation (r=0.14, p=0.21, **Figure 3E**). These results support the credibility of our pipeline integrating text-mining with miRNA prediction results.

We conducted enrichment analysis to explore the biological and pathological functions of the top miRNAs, determined by the average rank of frequency and degree. Surprisingly, the top five miRNAs and their target mRNAs were identical in both IBD and UC, leading to the same enrichment results (**Figure 3G**). The enriched KEGG terms were closely related to IBD pathology, although the top enrichments of the relative miRNAs displayed limited overlap. Specifically, targets of hsa-miR-146a were enriched in colorectal cancer and tight junctions, targets of hsa-miR-155 were enriched in the PD-L1 pathway and T cell receptor signaling pathway, targets of hsa-miR-21 were enriched in the JAK-STAT signaling pathway, cytokine-cytokine receptor interaction, and B cell receptor signaling pathway, targets of hsa-miR-223 were enriched in MAPK signaling and necroptosis, and targets of hsa-miR-34a were enriched in vascular smooth muscle contraction and inflammatory mediator regulation of transient receptor potential (TRP) channels in both IBD and UC (**Figure 3G**). In CD, hsa-miR-122 was functionally regulating the PI3K-Akt signaling pathway and gastric acid secretion, while hsa-miR-125a and hsa-miR-204 were functionally enriched in the MAPK signaling pathway (**Figure 3H**). Hsa-miR-155, hsa-miR-21, and hsa-miR-223 demonstrated a similar pattern among IBD, CD, and UC (**Figures 3G, H**).

### 3.3 Cellular sources of miRNAs in scRNA-seq datasets of IBDs

**Figure 4:**
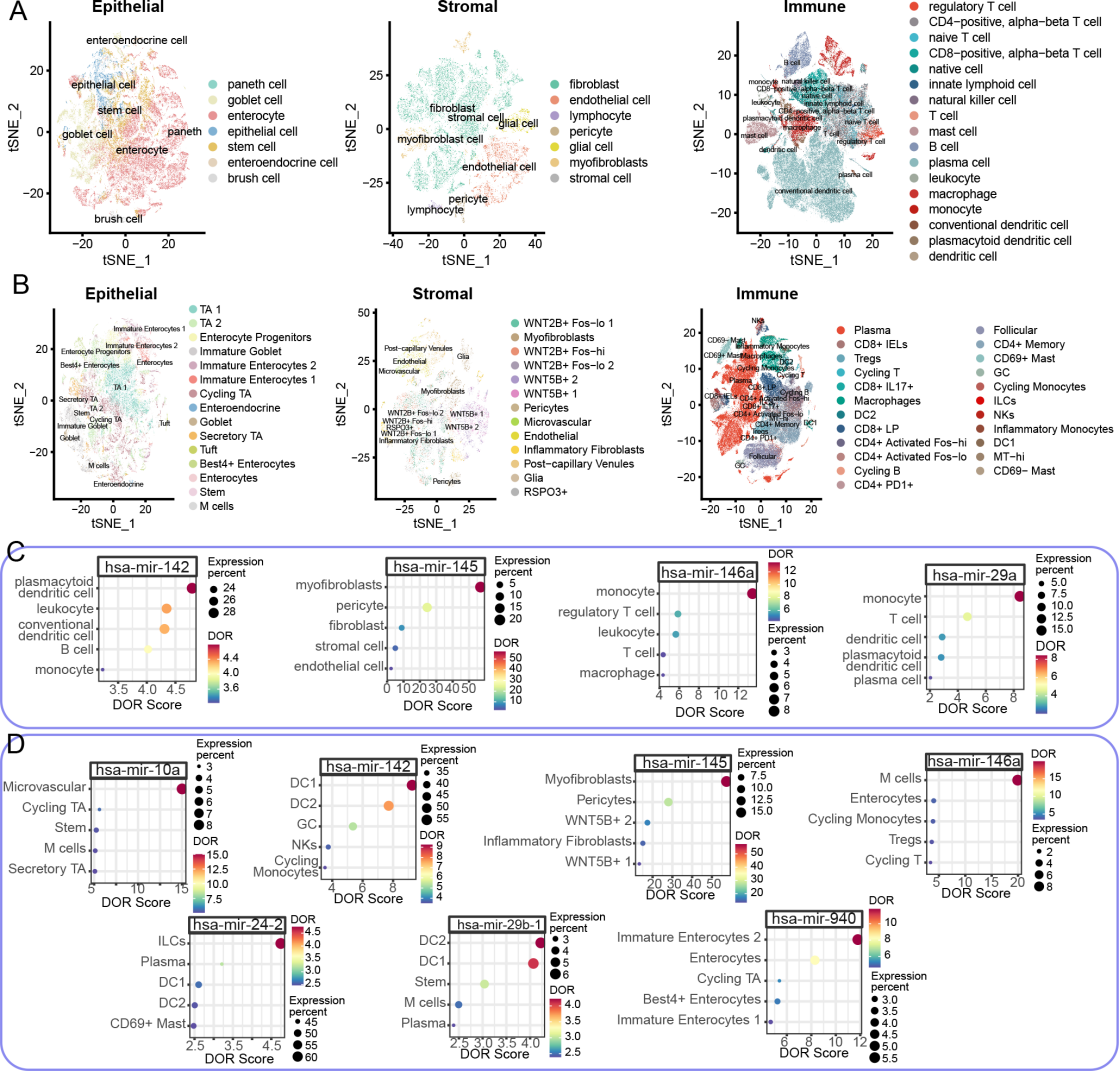
Cell type-specific miRNA expression profiles in large-scale CD and UC colon scRNA-seq datasets. **(A, B)** t-distributed Stochastic Neighbor Embedding (t-SNE) dimensionality reduction plots of epithelial (left), stromal (middle), and immune (right) cells in CD **(A)** and UC **(B)** colon scRNA-seq datasets. Colors indicate distinct cell types. **(C, D)** Dot plots showing the top five DOR scores for relevant miRNAs in CD **(C)** and UC **(D)** colon scRNA-seq datasets. Dot color represents the DOR score, while size indicates the expression percentage in respective cell types. The x axis represents the DOR score while the y axis represents the cell type.

We next examined the cellular sources of miRNAs by cross-analyzing two large-scale scRNA-seq datasets. DOR estimation was employed to examine the cell expression specificity of the tested miRNAs, considering the relative ratio of true to false expression levels of specific miRNAs. After quality control, normalization, clustering, and annotation, we analyzed a total of 97,788 epithelial cells, 39,433 stromal cells, and 152,509 immune cells in the CD scRNA-seq dataset across healthy control (Heal), non-inflammation (NonI), and inflammation (Infl) sites of CD colon tissues (**Figure 4A**). For UC cohorts, the numbers of epithelial, stromal, and immune cells were 81,082, 31,212, and 209,266, respectively (**Figure 4B**). The epithelial, stromal, and immune cells were further divided into subtypes according to marker genes and the original publications (**Figures 4A, B**).

To provide a comprehensive and informative cellular source of identified miRNAs, we merged the expression matrix of cell subtypes and estimated DOR. In the CD colon scRNA-seq dataset, five miRNAs were tested, while the number in UC cohorts was twenty. To reduce false positives in identifying the cellular source of specific miRNAs, miRNAs with less than 5% expression in cell subtypes were considered insignificant. Four miRNA-cell subtype pairs were identified in the CD colon scRNA-seq dataset (**Figure 4C**). Specifically, hsa-miR-142 was primarily expressed in plasmacytoid dendritic cells (pDCs), with additional expression in leukocytes and conventional dendritic cells (cDCs) (**Figure 4C**). Hsa-miR-145 was predominantly expressed in myofibroblasts, while hsa-miR-146a and hsa-miR-29a were enriched in monocytes (**Figure 4C**). In UC conditions, we also observed miRNA-cell subtype pairing. Hsa-miR-10a was enriched in microvascular cells, hsa-miR-142 in DC1, hsa-miR-145 in myofibroblasts, hsa-miR-146a in microfold cells (M cells), hsa-miR-23-2 in innate lymphoid cells (ILCs), hsa-miR-29b-1 in DC2, and hsa-miR-940 in immature enterocytes 2 (**Figure 4D**). Notably, hsa-miR-145 was consistently enriched in myofibroblasts in both CD and UC cohorts, highlighting the significant role of the hsa-miR-145-myofibroblast pair in IBD development. Moreover, despite the variation in cell subtypes, DCs were the major cellular source of hsa-miR-142 in both CD and UC cohorts (**Figures 4C, D**). Another shared miRNA in CD and UC, hsa-miR-146a, was enriched in different cell subtypes: monocytes in CD and M cells in UC (**Figures 4C, D**).

Interestingly, the expression levels of the identified miRNAs in their respective source cells also varied across disease states. In the CD colon scRNA-seq dataset, hsa-miR-142, hsa-miR-146a, and hsa-miR-29a were enriched in inflamed colon tissue, while hsa-miR-142 was more highly expressed in non-inflamed colon tissue (**Supplementary Figure S1A**). In the UC colon scRNA-seq dataset, hsa-miR-10a, hsa-miR-146a, hsa-miR-24-2, and hsa-miR-29b-1 demonstrated higher expression levels in inflamed lesions (**Supplementary Figure S1B**). Unlike in the CD cohort, hsa-miR-142 was enriched in non-inflamed DC1, and hsa-miR-145 was more enriched in healthy and non-inflamed colon tissue (**Supplementary Figures S1A, B**).

### 3.4 Molecular and functional alternation in miRNA source cells

**Figure 5:**
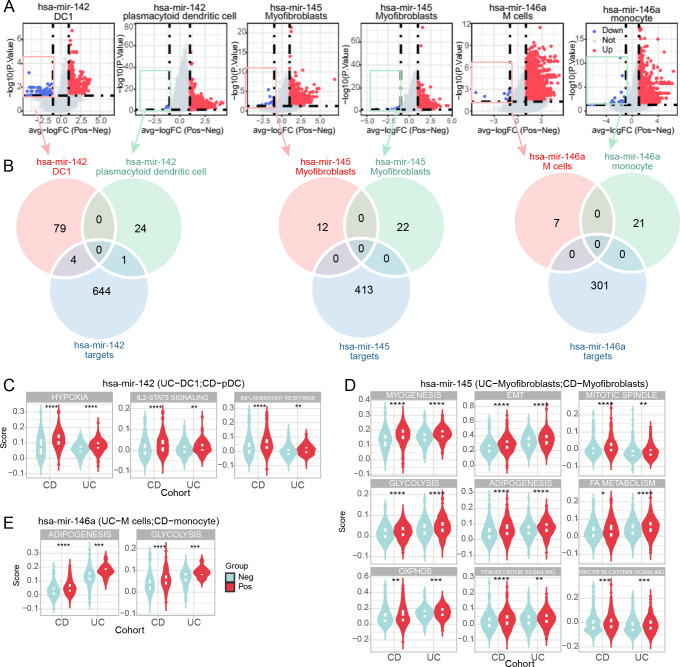
Molecular and functional alterations in miRNA-positive cell subclusters. **(A)** Volcano plots of differentially expressed genes (DEGs) between miRNA-positive and miRNA-negative cell subclusters in CD and UC scRNA-seq cohorts. Red and blue dots represent upregulated and downregulated genes, respectively, in miRNA-positive cell subclusters. **(B)** Venn diagrams illustrating the overlap between downregulated genes in miRNA-positive cell subclusters in CD and UC cohorts and predicted miRNA targets. **(C–E)** Violin plots depicting molecular functional signatures of miRNAs shared by CD and UC cohorts, including hsa-miR-142 **(C)**, hsa-miR-145 **(D)**, and hsa-miR-146a **(E)**. The color represents the positively expressed relative miRNA cell subcluster while the light blue represents the negatively expressed miRNA cell subcluster. *p<0.05; **p<0.01; ***p<0.001; ****p<0.0001.

To further clarify the miRNA-mRNA regulatory mechanism *in situ*, we examined the molecular and functional alterations of miRNA source cells at the single-cell level, both in CD and UC cohorts. We first divided the miRNA source cells into positive and negative subclusters based on the expression status of the relevant miRNA. Our initial hypothesis was that miRNAs function autonomously within local cells. Considering that miRNAs exert their function by interacting with and regulating their target genes, the down-regulated genes in miRNA-negative subclusters were considered candidates for miRNA targets. DEG analysis demonstrated 83 and 25 down-regulated genes in hsa-miR-142+ DC1 (UC cohort) and pDC (CD cohort), respectively (**Figure 5A**). For the hsa-miR-145 and myofibroblast pair, there were 12 and 22 down-regulated genes in UC and CD hsa-miR-145+ myofibroblasts, respectively (**Figure 5A**). Additionally, there were 7 and 21 down-regulated genes in hsa-miR-146+ M cells (UC cohort) and monocytes (CD cohort), respectively (**Figure 5A**).

Interestingly, we compared the down-regulated genes and text-mining identified target genes, finding limited overlap (**Figure 5B**). This result contradicted our initial hypothesis, indicating that the DOR-identified source cells were not the target cells of the relevant miRNAs. However, we did find some common signatures shared by the relative miRNA source cells, whether they were the same or different cell types. Specifically, both hsa-miR-142+ DC1 and pDC exhibited a hyper-inflammatory status, including hypoxia, IL2/STAT5 signaling, and inflammatory response (**Figure 5C**). The hsa-miR-145+ myofibroblasts in CD and UC cohorts were characterized by an enhanced mesenchymal phenotype, such as epithelial-mesenchymal transition (EMT) and myogenesis (**Figure 5D**). Additionally, hsa-miR-145+ myofibroblasts were in a proliferative state compared to the negative ones (**Figure 5D**). Enhanced metabolism was another characterization of hsa-miR-145+ myofibroblasts, including glycolysis, adipogenesis, fatty acid metabolism, and oxidative phosphorylation (OXPHOS) (**Figure 5D**). Additionally, activated PI3K/AKT/MTOR and WNT/β-catenin signaling were revealed in hsa-miR-145+ myofibroblasts (**Figure 5D**). Moreover, although varied in source cells, hsa-miR-146a was associated with enhanced adipogenesis and glycolysis in M cells and monocytes (**Figure 5E**). These results support that source cells share some molecular signatures and that miRNA effects can occur through a distal mechanism.

## 4 Discussions

This study presents a comprehensive analysis of miRNA-mRNA regulatory networks in IBDs, integrating text mining, miRNA target prediction, and single-cell RNA sequencing data. Our findings provide novel insights into the complex regulatory mechanisms of miRNAs in IBD pathogenesis, filling the gap in the cellular specific origin of miRNAs, highlighting potential targets for further investigation and therapeutic development.

### 4.1 Distinct miRNA profiles in IBD subtypes

Our text mining approach revealed distinct miRNA profiles between CD and UC, despite their shared inflammatory nature. The observation that top miRNAs showed more frequent overlap between IBD and UC than CD suggests that miRNAs might play different regulatory roles in these two major IBD subtypes. This finding aligns with previous studies indicating distinct pathophysiological mechanisms between CD and UC (38, 39). The lower frequency of both miRNAs and mRNAs identified in CD compared to UC highlights a potential gap in CD research, emphasizing the need for more focused studies on CD-specific molecular mechanisms.

### 4.2 Integrated miRNA-mRNA regulatory networks

The construction of IBD subtype-specific miRNA-mRNA regulatory networks provides a valuable resource for understanding the complex interactions in IBD pathogenesis. The significant correlation between text-mining mentioned frequency and the degree of regulatory network for miRNAs in both IBD and UC validates our approach of integrating text mining with miRNA target prediction. This integrated approach offers a more comprehensive view of miRNA functions in IBDs than either method alone.

### 4.3 Functional implications of key miRNAs

The enrichment analysis of top miRNAs revealed their involvement in critical pathways related to IBD pathology. For instance, the enrichment of hsa-miR-146a targets in colorectal cancer and tight junction pathways suggests its potential role in epithelial barrier dysfunction, a key feature of IBDs (40). Similarly, the involvement of hsa-miR-155 in PD-L1 and T cell receptor signaling pathways highlights its potential role in regulating immune responses in IBDs (41). Interestingly, the primary target gene enriched functions were distinct among the relative top key miRNAs though these functions were close to the IBD pathological processes. This discovery elucidates the intricate and pleiotropic regulatory mechanisms of miRNAs in IBD molecular pathogenesis, while concurrently delineating discrete functional specificities among diverse miRNA species. These findings provide a foundation for future functional studies to elucidate the specific roles of these miRNAs in IBD pathogenesis.

### 4.4 Cellular sources of miRNAs and their implications

Our trans-omics analysis of scRNA-seq revealed cell type-specific expression patterns of key miRNAs across different IBD conditions. These results fill the gap in the source cells of miRNA in IBD molecular pathology. The varying cellular sources of some miRNAs between CD and UC, such as hsa-miR-146a, further underscore the distinct molecular mechanisms at play in these IBD subtypes. One explanation of the heterogenous miRNA source cells was the distinct involved intestinal wall layers as well as predisposing sites between CD and UC. Specifically, CD affects the entire layers of intestine while UC affects the mucosal layer (9). Besides, the batch effects and subjects heterogenous between scRNA-seq datasets might give an explanation to the phenomena. The consistent enrichment of hsa-miR-145 in myofibroblasts in both CD and UC cohorts suggests a crucial role for this miRNA-cell type pair in IBD development, possibly through regulation of fibrosis and tissue remodeling (41, 42).

### 4.5 Unexpected findings and new hypotheses

Intriguingly, our analysis of down-regulated genes in miRNA-positive cell subclusters revealed limited overlap with predicted miRNA targets. This unexpected finding challenges the initial hypothesis that miRNAs primarily act autonomously in their source cells. Instead, it suggests a more complex regulatory mechanism, possibly involving paracrine or endocrine-like effects of miRNAs (43, 44). This observation opens new avenues for research into the intercellular communication roles of miRNAs in IBD pathogenesis.

### 4.5 Functional alterations in miRNA source cells

The identification of common signatures in miRNA source cells, regardless of cell type, provides insights into the broader functional implications of these miRNAs. For example, the association of hsa-miR-142 positive DCs with a hyper-inflammatory status across different cell types suggests a conserved role for this miRNA in regulating inflammatory responses in IBDs. The hsa-miR-142+ DCs were signatured as pro-inflammation phenotype, including IL2/STAT5 signaling. The IL2/STAT5 signaling pathway plays a critical role in IBD progression, such as maintaining gut epithelial integrity and promoting regulatory T cells (45, 46). However, unwanted T cell activation was also observed at higher doses of IL2 (45). Consistent with the previous research, hsa-miR-142 was enhanced in IBDs and functional as a pro-inflammatory mediators (47). Similarly, the enhanced mesenchymal phenotype and metabolic alterations observed in hsa-miR-145 positive myofibroblasts highlight potential mechanisms by which this miRNA might contribute to tissue remodeling and fibrosis in IBDs (48, 49).

Myofibroblasts play a crucial role in normal tissue repair processes across various organs, but their abnormal hyper-activation can lead to fibrosis. During normal tissue repair, myofibroblasts operate in a dynamic process induced by tissue damage signals, such as inflammatory mediators and TGF-β. This process includes proliferation, fibroblast-to-myofibroblast transition, and epithelial-to-mesenchymal transition (EMT), followed by subsequent elimination via apoptosis (50). However, the chronic presence and continued activity of myofibroblasts characterize many fibrotic pathologies, which are common complications in IBDs (52). The TGF-β/Smad signaling pathway is considered vital in the development of fibrosis in multiple organs. In addition to the canonical TGF-β/Smad signaling pathway, TGF-β can activate other pathways, including extracellular regulated protein kinases (ERK), phosphatidylinositol-3-kinase/protein kinase B (PI3K/AKT), and WNT signaling pathways (53). Our results demonstrate that myofibroblasts are the primary cellular source of hsa-miR-145. Moreover, hsa-miR-145+ myofibroblasts are characterized by enhanced tissue repair and metabolic activity. Consistent with previous research, our results indicate that hsa-miR-145+ myofibroblasts exhibit higher activation scores for PI3K/AKT and WNT pathways (48). Previous studies have shown that hsa-miR-145 can reduce intestinal permeability and suppress IBD progression through the SOX9-CLDN8 pathway (51). Our findings bridge the gap between hsa-miR-145 and cellular interactions.

### 4.6 Limitations and future directions

While our study provides comprehensive insights into miRNA-mRNA regulatory networks in IBDs, several limitations should be addressed in future research. First, the reliance on text mining and predicted miRNA-mRNA interactions necessitates experimental validation of key findings. Second, the single-cell RNA sequencing data, while informative, represents a snapshot of cellular states and may not capture the full dynamics of miRNA regulation over the course of disease progression. Longitudinal studies and functional experiments are needed to fully elucidate the roles of identified miRNAs in IBD pathogenesis. The future work should focus on the experimental validation of key miRNA-mRNA interactions and their functional consequences in relevant cell types, independent datasets to verify the reproducibility and generalizability of the miRNA-mRNA regulatory networks, and nnockdown or overexpression studies in relevant cell lines or animal models can elucidate the biological functions and pathways associated with the key miRNAs. Investigation of the potential paracrine or endocrine-like effects of miRNAs in IBD pathogenesis. Development and testing of miRNA-based therapeutic approaches targeting the identified regulatory networks. Exploration of the potential of cell type-specific miRNA expression patterns as biomarkers for IBD diagnosis, prognosis, or treatment response.

### 4.7 Unexpected findings and new hypotheses

Intriguingly, our analysis of down-regulated genes in miRNA-positive cell subclusters revealed limited overlap with predicted miRNA targets. This unexpected finding challenges the initial hypothesis that miRNAs primarily act autonomously in their source cells. Instead, it suggests a more complex regulatory mechanism, possibly involving paracrine or endocrine-like effects of miRNAs (43, 44). This observation opens new avenues for research into the intercellular communication roles of miRNAs in IBD pathogenesis.

## 5 Conclusion

In conclusion, this study provides a comprehensive landscape of miRNA-mRNA regulatory networks in IBDs, integrating multiple layers of molecular and cellular data. Our findings not only enhance our understanding of the complex regulatory mechanisms underlying IBD pathogenesis but also highlight potential targets for novel diagnostic and therapeutic strategies. The unexpected observations regarding miRNA cellular sources and target gene relationships open new avenues for research into the diverse roles of miRNAs in intercellular communication within the context of chronic inflammatory diseases.

## Data Availability

CD and UC scRNA-seq datasets were obtained from the Single Cell Portal (https://singlecell.broadinstitute.org/single_cell) using accession numbers SCP1884 and SCP259, respectively. Comprehensive details regarding the scRNA-seq data, including clinical sample information and sequencing protocols, are available in the original source studies (33,34). Both datasets were processed using the same criteria. Epithelial, stromal, and immune cells were downloaded and subjected to scRNA-seq analysis.

## Supplementary material

Supplementary Figure 1 Differential expression of miRNAs in source cells across CD and UC disease states. **(A, B)** Expression levels of miRNAs across disease states in their respective source cells for CD **(A)** and UC **(B)**. Color intensity represents the average expression level of miRNAs in source cells, while dot size indicates the percentage of cells expressing the miRNA. The x axis represents the disease states including healthy control (Heal), non-inflammation (NonI), and inflammation (Infl).
