# Supplementary material for: The landscape of miRNA-mRNA regulatory network and cellular sources in inflammatory bowel diseases: insights from text mining and single cell RNA sequencing analysis
Source: Front Immunol. 2024 Aug 22;15:1454532. doi: 10.3389/fimmu.2024.1454532 (PMC11374595; doi:10.3389/fimmu.2024.1454532)
Supplement: Supplementary Figure 1 — Differential expression of miRNAs in source cells across CD and UC disease states. (A, B) Expression levels of miRNAs across disease states in their respective source cells for CD (A) and UC (B). Color intensity represents the average expression level of miRNAs in source cells, while dot size indicates the percentage of cells expressing the miRNA. The x axis represents the disease states including healthy control (Heal), non-inflammation (NonI), and inflammation (Infl). [file Datasheet1.pdf]

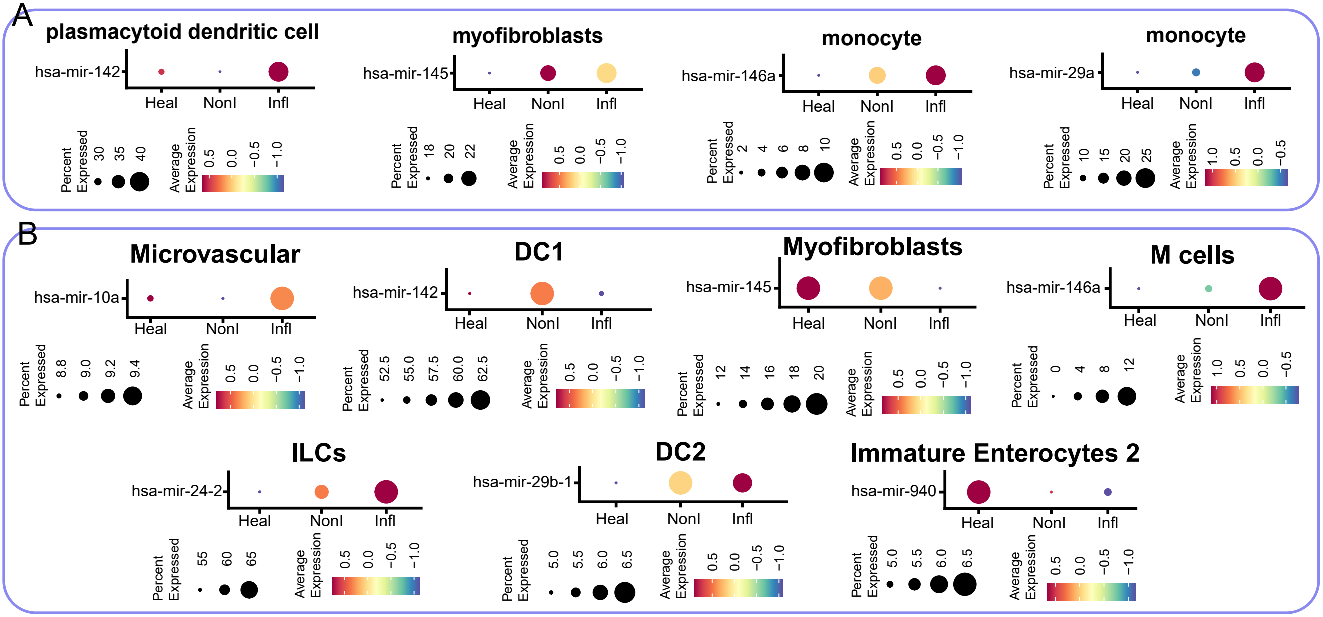
A
plasmacytoid dendritic cell
hsa-mir-142
myofibroblasts
hsa-mir-145
monocyte
hsa-mir-146a
monocyte
hsa-mir-29a
Heal
NonI
Infl
Percent Expressed
Average Expression
B
Microvascular
hsa-mir-10a
DC1
hsa-mir-142
Myofibroblasts
hsa-mir-145
M cells
hsa-mir-146a
ILCs
hsa-mir-24-2
DC2
hsa-mir-29b-1
Immature Enterocytes 2
hsa-mir-940
